# Cardiorespiratory effects of venous lipid micro embolization in an experimental model of mediastinal shed blood reinfusion

**DOI:** 10.1186/1749-8090-4-48

**Published:** 2009-09-15

**Authors:** Atli Eyjolfsson, Ignacio Plaza, Björn Brondén, Per Johnsson, Magnus Dencker, Henrik Bjursten

**Affiliations:** 1 Department of Cardiothoracic Surgery, Department of Clinical Sciences, Lund University, Sweden; 2 Department of Anaesthesiology, Department of Clinical Sciences, Lund University, Sweden; 3 Department of Clinical Physiology, Department of Clinical Sciences, Malmö, Lund University, Sweden

## Abstract

**Background:**

Retransfusion of the patient's own blood during surgery is used to reduce the need for allogenic blood transfusion. It has however been found that this blood contains lipid particles, which form emboli in different organs if the blood is retransfused on the arterial side. In this study, we tested whether retransfusion of blood containing lipid micro-particles on the venous side in a porcine model will give hemodynamic effects.

**Methods:**

Seven adult pigs were used. A shed blood surrogate containing 400 ml diluted blood and 5 ml radioactive triolein was produced to generate a lipid embolic load. The shed blood surrogate was rapidly (<2 minutes) retransfused from a transfusion bag to the right atrium under general anesthesia. The animals' arterial, pulmonary, right and left atrial pressure were monitored, together with cardiac output and deadspace. At the end of the experiment, an increase in cardiac output and pulmonary pressure was pharmacologically induced to try to flush out lipid particles from the lungs.

**Results:**

A more than 30-fold increase in pulmonary vascular resistance was observed, with subsequent increase in pulmonary artery pressure, and decrease in cardiac output and arterial pressure. This response was transient, but was followed by a smaller, persistent increase in pulmonary vascular resistance. Only a small portion of the infused triolein passed the lungs, and only a small fraction could be recirculated by increasing cardiac output and pulmonary pressure.

**Conclusion:**

Infusion of blood containing lipid micro-emboli on the venous side leads to acute, severe hemodynamic responses that can be life threatening. Lipid particles will be trapped in the lungs, leading to persistent effects on the pulmonary vascular resistance.

## Background

Autotransfusion of blood is used in surgical procedures to reduce the need for allogenic blood transfusion. The main reasons for doing this are to reduce costs and transfusion-related morbidity. Adverse effects of heterologous transfusions have recently been highlighted [1]. For example, it has been shown in cardiac surgery that heterologous blood transfusion may have negative effects on long-term survival [2,3].

In addition to autotransfusion, blood conservation strategies are employed routinely in several surgical procedures. In cardiac surgery, for example, blood lost in the pericardium or pleurae is routinely retransfused directly to the patient via the heart-lung machine. Sometimes, a centrifugal-based cell-washing procedure is used.

However, autologous transfusions in conjunction with surgery have raised some controversy, especially after the finding that this blood contains lipid particles [4-10]. Lipid particles have been found as emboli in many organs, including the brain and kidneys, after arterial retransfusion [6,11]. It has been suggested that lipid emboli contribute to organ dysfunction after surgery [12,13]. However, present methods of removing these emboli, such as filters and centrifuges, only seem to reduce the embolic load to a limited degree [4,8,13,14], and no safe and truly efficient way of removing these lipid particles before retransfusing shed blood is available. It has been suggested that one way of dealing with the problem could be to transfuse shed blood on the venous side, utilizing a postulated filtering effect of the lungs.

Several groups have studied the pathologic effect of large lipid emboli in the venous circulation in conjunction with orthopedic surgery, and found adverse hemodynamic and respiratory effects [15-17]. However, little is known about the effect that numerous lipid micro-emboli, as found in shed blood collected from the pericardium during cardiac surgery, may have on the pulmonary circulation.

In this study we investigated the effect of re-transfusion of blood containing lipid micro-emboli on the venous side, in terms of hemodynamic and respiratory effects, as well as the lipid removal capacity of the lungs in a porcine model.

## Methods

### Study protocol

**Figure 1:**
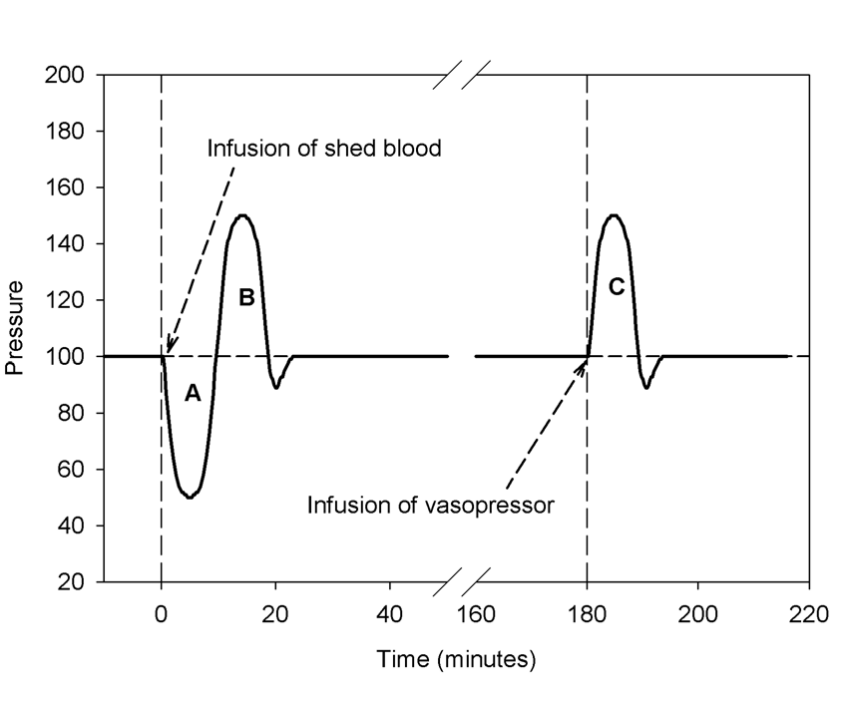
**The protocol used for determining physiological response**. The vertical dashed lines indicate the infusion of the shed blood surrogate, and the infusion of epinephrine and Ringer's lactate. A denotes the Area Under the Curve (AUC) as a measure of decline in blood pressure over time. B denotes the AUC indicating blood pressure increase. C denotes the increase in blood pressure after the period of increased blood pressure and flow induced with epinephrine and Ringer's lactate.

After approval from the regional animal study ethics committee, 7 adult pigs were prepared. The animals (70 kg) were anesthetized and mechanically ventilated. When the animals showed circulatory stability, a 10 minute resting period without any activity or stimulation was instituted. After this period, a shed blood surrogate containing lipid micro-emboli was infused according to the protocol illustrated in Figure 1. The animals were monitored for the following three hours, after which cardiac output and pulmonary pressure were increased by infusing 200 μg of epinephrine (Adrenaline Merck NM, Merck NM, Stockholm, Sweden) together with 500 ml Ringer's lactate (Fresenius Kabi, Uppsala, Sweden).

### Monitoring

Catheters for monitoring, drug delivery and blood sampling were inserted into a vein in one of the ears, the jugular internal vein, the left and right atrium, the pulmonary artery and the femoral and carotid arteries. During the experiment, arterial and pulmonary blood pressure, central venous and left atrium pressure, pulse, nasopharyngeal temperature, ventilator settings and pulse oximetry were monitored continuously with a SpaceLabs Medical monitoring system (SpaceLabs, Issaquah, WA, USA). Cardiac output was recorded with a Transonic HT207 ultrasonic flow meter (Transonic System Inc., Ithaca, New York, USA) or a Cardiomed CM 4000 transit time ultrasound flow meter (Cardiomed, Toronto, Canada). A 14 mm or 16 mm probe was used to measure the cardiac output in the pulmonary artery. Complete monitoring data were obtained from all animals except one. The recordings of pulmonary artery pressure in one animal were unstable due to deficient contact between the ultrasonic transducer and the pulmonary artery, and this pressure was excluded from the analysis.

### Anesthesia

Premedication was performed with an intramuscular injection of 15 mg/kg ketamine chloride (Ketalar^®^, Pfizer Inc., New York, NY, USA) and 0.2 mg/kg xylasine (Rompun^®^, Bayer, Gothenburg, Sweden). Induction and maintenance of anesthesia were achieved using an infusion of 0.15 mg/kg/min ketamine chloride and 0.01 mg/kg/min pancuronium bromide (Pavulon^®^, N.V. Organon, Oss, the Netherlands), or an infusion of 0.1-0.2 mg/kg/min propofol (Diprivan^®^, Astra-Zeneca, Sweden), together with intermittent injections of fentanyl (Leptanal^®^, Lilly, France) and atracrium besylate (Tracrium^®^, Glaxo, Täby, Sweden). The different anaesthetic protocols were due to a change in laboratory practice for other reasons, and not related to this study.

The animals underwent tracheostomy and were connected to a ventilator (Siemens Servo 900C, Solna, Sweden). Volume controlled ventilation was applied with 50% FiO_2_. Arterial blood was drawn for blood gas sampling at the start of the study, immediately before administration of radiolabelled triolein, 5, 15, 30 and 45 minutes thereafter, and immediately before the period of increased pressure, and 5, 15, 30 and 45 minutes thereafter. Exhaled gases were monitored continuously with a NiCO or CO2SMO Plus respiratory profile meter (Novametrix Medical Systems Inc., Norwell, MA, USA). The dead space was calculated using the Bohr equation principle from blood gases and exhaled CO_2 _[18].

### Experimental procedure

A sternotomy was performed to expose the heart and 400 IU/kg heparin (LEO Pharma A/S, Copenhagen, Denmark) was administered before starting the experiment. At the end of the experiment the animals were sacrificed using potassium chloride (B. Braun, Melsungen, Germany) and thiopental sodium (Pentothal^®^, Abbot, Chicago, IL, USA).

### Administration of radiolabelled triolein

Radioactive triolein (Amersham BioSciences, Little Chalfont, UK) was mixed with 65% non-radioactive triolein solution (Carl Roth GmbH., Karlsruhe, Germany). The proportions used were such that 5 ml of the final solution contained 1 mCi of radioactivity.

A shed blood surrogate was then produced by mixing 200 ml arterial blood with 200 ml saline and 5 ml of the 1 mCi radioactive triolein solution. This will yield approximately 1% lipid content, and has been used in similar studies [6,19]. The surrogate was gently agitated for approximately five minutes and retransfused from a pressurized transfusion bag with a 40-micron filter in the infusion aggregate, into the animal via the venous line during a 2-minute period.

### Blood sampling

Blood samples for the determination of radioactivity were drawn from a separate catheter in the carotid artery at baseline, at the start of infusion of the shed blood surrogate, every minute for fifteen minutes and then every ten minutes up to 3 hours after infusion.

When the infusion of adrenaline and 500 ml Ringer's lactate was started, blood was sampled at the start of infusion, every minute for fifteen minutes and then every ten minutes up to one hour after the infusion. On each occasion 0.2 ml blood was collected for the determination of radioactivity.

### Sample preparation

To each sample of blood, 2 ml Soulene-350^® ^(Packard Bioscience, Groningen, the Netherlands) was added to dissolve the cells. The sample was left in an air heater at 37°C overnight. To decolorize the samples, 0.2 ml hydrogen peroxide was added twice, with overnight incubation at 37°C between. One ml 95% ethanol was added, followed by 15 ml scintillation fluid (Hionic Fluor, Packard Bioscience, Groningen, the Netherlands) [6]. The samples were then left to rest for 4-6 days in order for the chemoluminescence to decrease.

Scintillation counting was performed with a liquid scintillation counter (14814 Win Spectral Guardian, Wallac Oy, Turku, Finland). The specific activity of tritium was calculated for each sample. Two separate measurements were performed, and the mean value of the two measurements was used. Radioactivity is reported as the number of disintegrations per minute per ml (DPM/ml).

### Statistics

All values are expressed as the mean ± 1 standard deviation (SD). Comparisons between groups were made with a two-tailed student's t-test, unless otherwise stated. To quantify the physiological response in terms of a decrease or increase in blood pressure, the area under the curve (AUC) was calculated from the period of blood pressure change (Figure 1). The end of the change was defined as the point of time when the blood pressure had returned to the pre-event value.

## Results

Infusion of the shed blood surrogate resulted in an almost immediate increase in the pulmonary pressure. In one animal, the infusion led to total circulatory collapse within 10 minutes due to acute right heart failure, despite attempts to reverse the condition with epinephrine. This animal was excluded from the analysis. Thus, the results from 6 animals are presented.

### Infusion of shed blood surrogate

**Figure 2:**
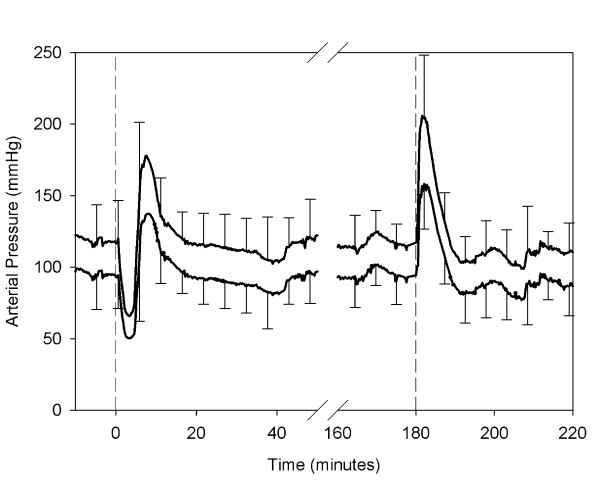
**Systolic and diastolic arterial blood pressure**. Mean values ± 1SD during the experiment. The first dashed line indicates the infusion of the shed blood surrogate. The second dashed line indicates the infusion of epinephrine and Ringer's lactate.

The response of the arterial blood pressure after infusion of shed blood was biphasic (Figure 2). Five of the 6 animals exhibited an initial decline in arterial blood pressure, followed by an increase in pressure. This initial decrease in systolic blood pressure was 53 ± 39 mmHg from baseline, and was recorded after a mean of 158 ± 51 seconds. The subsequent increase in systolic blood pressure from pre-infusion baseline was 70 ± 69 mmHg. The physiological response measured as the AUC for the decreasing period (Figure 1A) was significantly different from no response (p < 0.05). The response expressed as the AUC for the increase in arterial pressure did not reach significance (p < 0.10) (Figure 1B).

**Figure 3:**
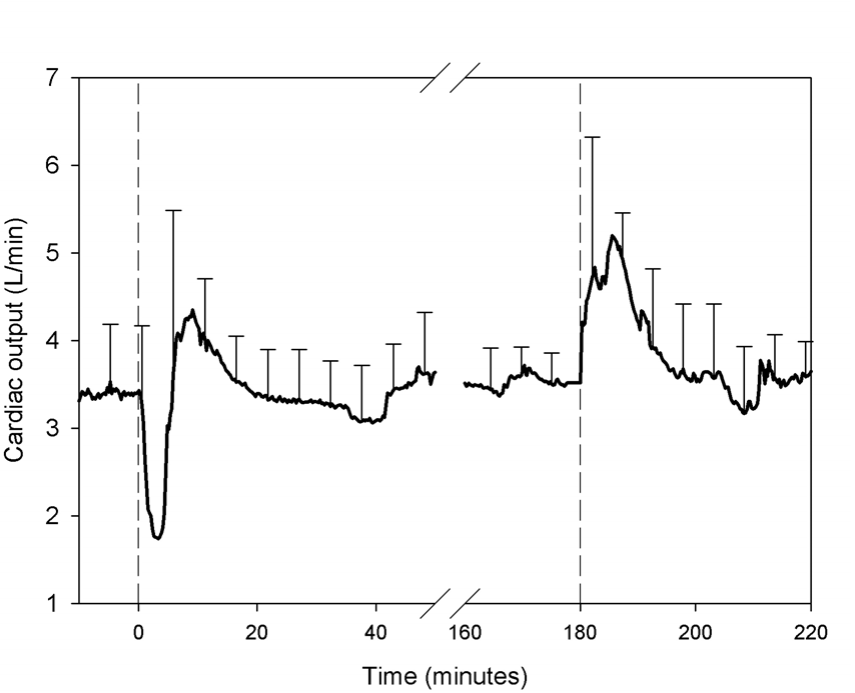
**Cardiac output**. Mean values ± 1SD during the experiment. The first dashed line indicates the infusion of shed blood surrogate. The second dashed line indicates the infusion of epinephrine and Ringer's lactate.

**Figure 4:**
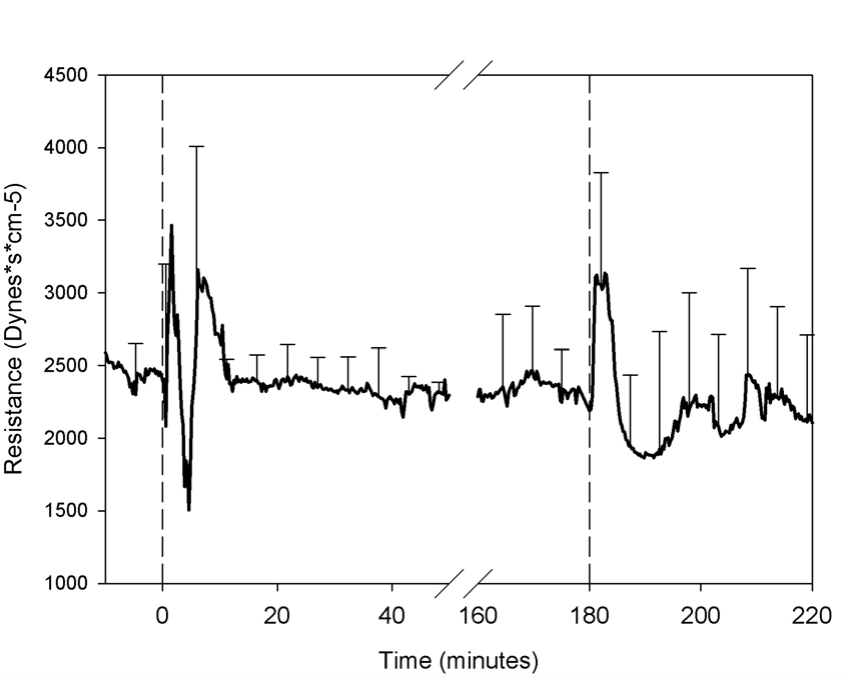
**Peripheral vascular resistance**. Mean peripheral vascular resistance as determined from arterial blood pressure and cardiac output. The first dashed line indicates the infusion of shed blood surrogate. The second dashed line indicates the infusion of epinephrine and Ringer's lactate.

Cardiac output declined concomitantly with the decrease in arterial pressure (Figure 3), from 3.39 ± 0.68 to 1.59 ± 1.95 L/minute (p < 0,001) and was on average 53% from base-line. The changes in systemic vascular resistance (SVR) varied from animal to animal. In 2 animals there was almost no response. In the other animals there were both rapid increases and decreases in SVR during the initial period of hemodynamic instability, but no pattern could be discerned (Figure 4).

**Figure 5:**
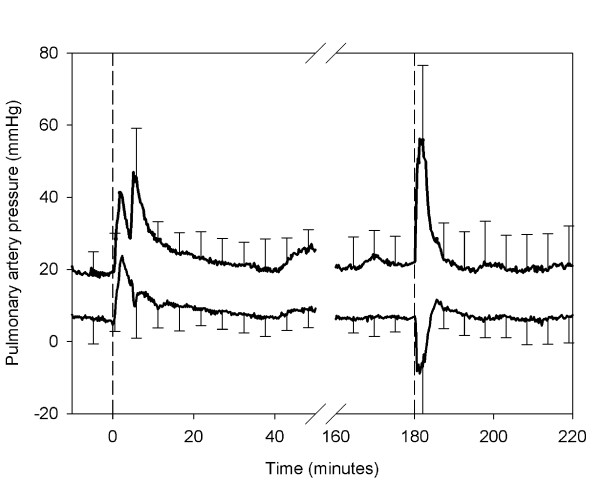
**Systolic and diastolic pulmonary blood pressure**. Mean systolic and diastolic pulmonary blood pressure ± 1SD during the experiment. The first dashed line indicates the infusion of shed blood surrogate. The second dashed line indicates the infusion of epinephrine and Ringer's lactate.

The response of the pulmonary pressure after infusion of the shed blood was biphasic (Figure 5). All animals showed an initial increase in pulmonary pressure, followed by a short decrease before a second rapid increase. The initial increase in systolic pulmonary pressure from baseline was 36 ± 10 mmHg (p < 0.05), which represents a 156% increase in pulmonary systolic pressure. The secondary increase in pulmonary pressure was 47 ± 17 mmHg (p < 0.05) above baseline.

**Figure 6:**
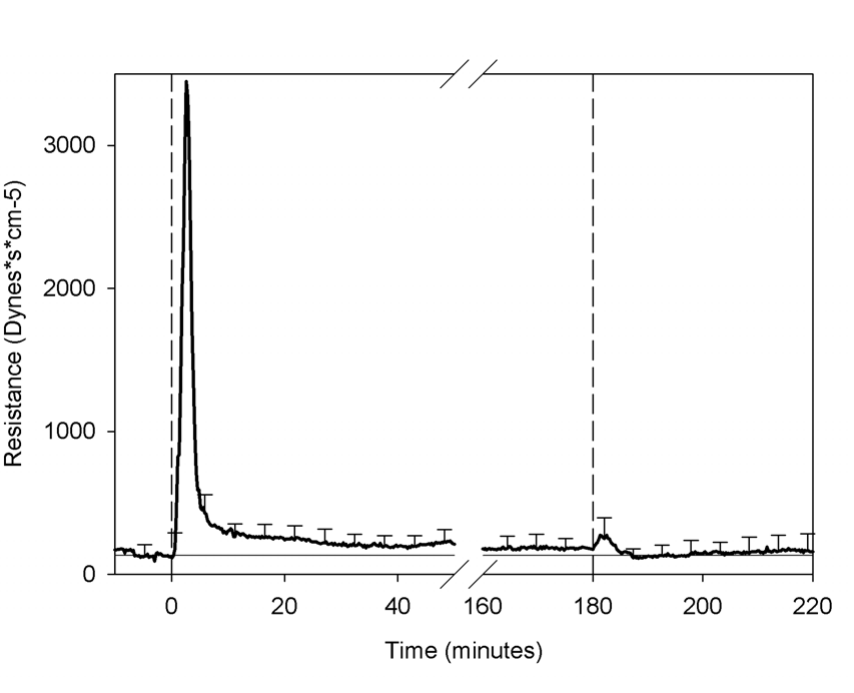
**Pulmonary vascular resistance**. Mean pulmonary vascular resistance as determined from arterial blood pressure and cardiac output. The first dashed line indicates the infusion of shed blood surrogate. The second dashed line indicates the infusion of epinephrine and Ringer's lactate. The horizontal line denotes the baseline value calculated from the PVR during the 10-minute resting period (prior to the shed blood surrogate infusion).

Pulmonary vascular resistance (PVR) increased significantly in all animals (Figure 6). The mean increase in PVR from 116 ± 67 dynes * s * cm^-5 ^before infusion to 3446 ± 3676 dynes * s * cm^-5 ^at the maximum PVR, and did not return completely to baseline until after the infusion of epinephrine and Ringer's lactate (Figure 6).

**Figure 7:**
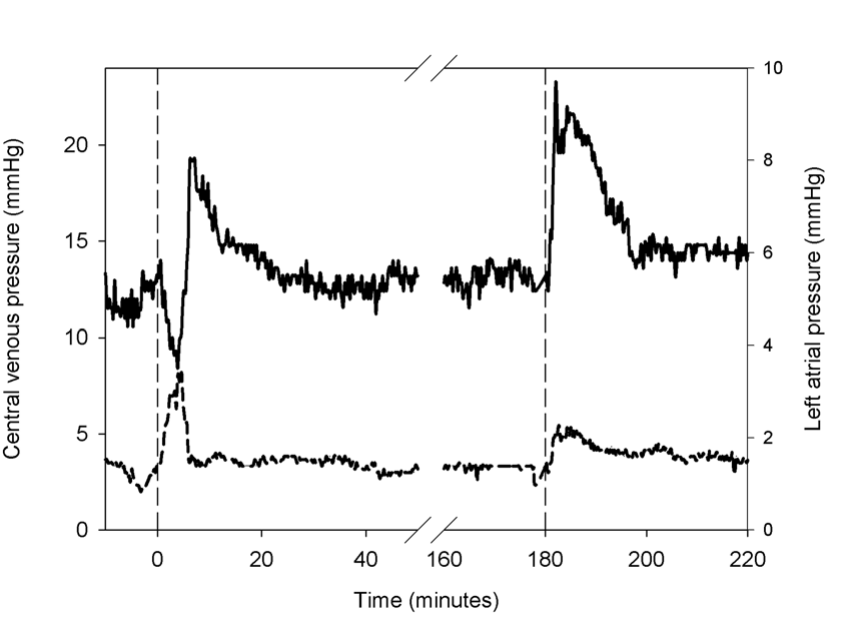
**Central venous pressure and left atrial pressure**. Mean central venous pressure (dashed line) and left atrial pressure (solid line). The first dashed vertical line indicates the infusion of shed blood surrogate. The second dashed vertical line indicates the infusion of epinephrine and Ringer's lactate.

The central venous pressure increased and the left atrial pressure decreased, in response to the infusion of the shed blood surrogate (Figure 7).

### Effects of the pharmacologically increased blood flow and pressure

After infusion of epinephrine and Ringer's lactate an increase in arterial blood pressure ensued (Figure 2), as shown by a significant increase in the AUC of the blood pressure (p < 0.001). In addition, there was an increase in cardiac output and SVR (Figures 3 and 4).

Pulmonary artery pressure and PVR increased transiently after the infusion of epinephrine and Ringer's lactate.

### Levels of radioactivity

**Figure 8:**
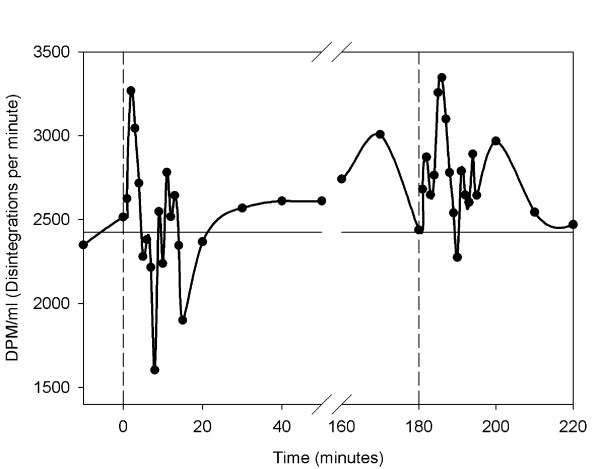
**Radioactivity**. Mean amount of radioactivity in the carotid artery at each sampling time, as a measure of the amount of emboli passing through the pulmonary circulation. The first dashed line indicates the infusion of shed blood surrogate. The second dashed line indicates the infusion of epinephrine and Ringer's lactate. The horizontal line denotes the baseline value calculated from the two samples taken before infusion.

The radioactivity levels in arterial blood increased after the infusion of the shed blood surrogate (Figure 8). From the baseline level of 2369 ± 1164 DPM/ml levels increased to a peak of 3953 ± 1532 DPM/ml (p < 0.05), at a mean time of 100 ± 50 seconds after infusion.

After the period of increased pulmonary blood flow and pressure, the peak level was 4080 ± 981 DPM/ml at a mean time of 390 ± 112 seconds, and was significantly higher than the baseline value of 2369 ± 1164 DPM/ml (p < 0.05).

### Capnography

**Figure 9:**
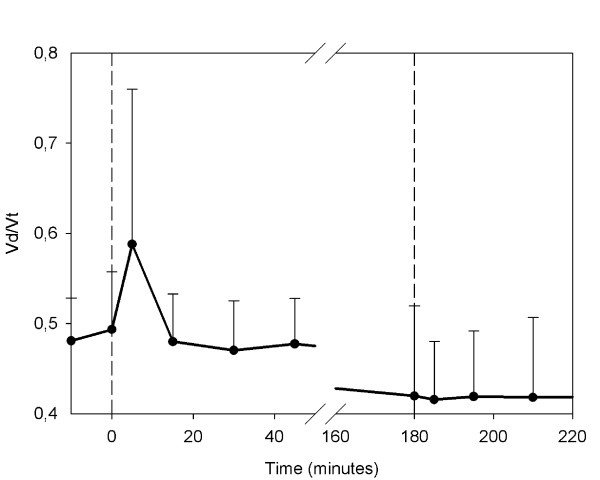
**Ventilatory deadspace**. Mean ventilatory deadspace estimated from blood gas analysis and capnography. The first dashed line indicates the infusion of shed blood surrogate. The second dashed line indicates the infusion of epinephrine and Ringer's lactate.

The deadspace (Vd/Vt) increased after infusion of the shed blood surrogate (Figure 9), and reached its maximal levels after 5, 15 or 30 minutes in 5 of the 6 animals. In one animal, no change in deadspace was observed. The mean level of Vd/Vt before infusion of the shed blood was 0.49 ± 0.06, compared to the highest levels after infusion 0.61 ± 0.15 (p = 0.06).

## Discussion

This study clearly demonstrates that a rapid intravenous infusion of blood laden with lipid micro-particles has significant hemodynamic effects in a porcine model, with the most obvious finding being a considerable increase in PVR and subsequent hemodynamic changes. Normally an infusion of volume would yield and increased right ventricular filling and subsequent increase in cardiac output[20]. The results also suggest that a substantial fraction of the lipid micro-embolic load is trapped in the pulmonary vasculature.

In addition to the obvious acute hemodynamic changes directly after the infusion of shed blood, we also observed hemodynamic effects of moderate duration. The acute increase in PVR led to both right ventricle failure and decreased cardiac output and, as a consequence, reduced arterial blood pressure. These hemodynamic changes could be attributed to the increase in pulmonary vascular resistance.

The finding that infusing lipid material on the venous side leads to negative hemodynamic consequences is not new. The phenomenon has been studied extensively in studies addressing adult respiratory distress syndrome and lipid embolization in orthopedic trauma [15,16,21-24]. All these studies, however, used models of macro-embolization, where the infused lipid was normally a single bolus of pure triolein, thus forming one or more large particles. The abrupt hemodynamic changes found in our study was similar to that in models of larger emboli [21,23]. In our model, the intention was to simulate the situation of re-infusing shed blood collected during surgery, which is rich in lipid micro-emboli. A shed blood surrogate was therefore produced, containing radiolabelled triolein, which was agitated well to disperse the lipid into smaller particles to mimic the clinical situation. In addition, the infusion was passed through a 40 μm infusion filter to disperse the particles even more. The particles would therefore theoretically be able to migrate deeply into the capillaries of the lung.

The acute changes were obvious, with more than a 20-fold increase in PVR. The mechanisms causing this increase were not explored in this study. However, the increase was transient in nature, and it could therefore be speculated that several mechanisms play a role in this rapidly rising PVR. Mechanical obstruction of capillaries can play a role in the pathophysiology. A vascular wall response, leading to a spasm, could also be involved; triolein itself may have triggered such a spasm. Several authors have suggested that liberated free fatty acids have direct local toxic effects, which could promote a vasospasm [16,25]. Whatever direct effects the triolein may have had, liberated free fatty acids from the infused triolein could further have aggravated the response. The acute response after the infusion of the shed blood may well be multifactorial, and the different mechanisms considered here additive.

Changes of moderate duration were seen throughout the 3 hours of the test, but were not as striking. The PVR remained slightly elevated until epinephrine and Ringer's lactate were infused (Figure 5). Consequently, the pulmonary artery pressure did not return to baseline at the same time as the other parameters (Figure 6). Therefore, at least one of the mechanisms behind the acute increase in PVR acts over a sustained period of time. It can not be determined from the results of this study which mechanism is responsible for the delayed increase. However, mechanical obstruction by the lipid emboli seems plausible.

The second part of the experiment involving a pharmacologically induced increase in cardiac output and pulmonary pressure, was carried out to study the effects of an increased pressure gradient on the lipid emboli wedged in the lungs. From the hemodynamic data, it can easily be concluded that the response intended, in terms of hemodynamic changes, was achieved. The PVR increased immediately with an increase in both arterial and pulmonary pressure. After this increase in PVR, there was a period of decreased mean PVR, compared to levels before increased blood pressure. The PVR then slowly increased once more. These changes did not, however, reach statistical significance, probably due to the small number of animals. Our interpretation is that this increase in pressure either wedged the particles further down into the capillaries or forced some of them to pass out of the capillaries of the lungs and into the systemic circulation.

Radiolabelled triolein has previously been used in a study to determine the differential distribution of lipid emboli after an arterial infusion [6]. The embolic load could easily be characterized using measurements of the radioactivity. The levels found in the present study, were low in comparison with that study, and had a large variation. However, a significant increase in PVR was observed directly after the infusion, after which levels seemed to return to baseline values (Figure 6). After the period of induced increase in cardiac output, radioactivity levels increased significantly, and thus some of the trapped emboli must have been forced out of the lungs. Sikorski et al concluded that 95% of triolein infused as macro-emboli was trapped in the lungs [15]. Our findings suggest that a high proportion of micro-emboli will also be trapped in the lungs.

The shed blood surrogate, consisting of 200 ml saline, 200 ml blood and 5 ml triolein, was used to mimic the clinical situation of retransfusing shed blood, collected from the operating wound. The true composition of lipid particles in such blood has not been studied thoroughly. In this model, triolein was used, since it is the most common triglyceride in adipose tissue and represents 50% of the triglycerides[26]. A chemically more representative composition of triglycerides could affect results, but probably only in terms of local toxic effects. It could be argued that the effects of the transfusion of the surrogate are dose-dependent, and that the amount of lipids given is experimental and not representative of the clinical setting. However, experience from a previous study shows that the dose used results in similar lipid droplet formation as seen on the surface of shed mediastinal blood [6]. For the moment, surprisingly few attempts have been made to characterize the lipid content of shed blood, and compare it with levels found in for instance orthopaedic surgery. One study estimated the lipids concentration in shed blood on average 0,4% in 400 ml blood[27]. Our model contained 1% and would therefore represent blood with lipid content in the high ranges. The shed blood surrogate was infused as a short bolus, to achieve a distinct effect with immediate response that could be correlated with the intervention. This is seldom the practice in the clinical setting. On the other hand, young healthy animals with uncompromised lung and heart function were used, which is seldom the case with patients, especially in cardiac surgery patients, in which both the pulmonary and right ventricle function may be compromised after cardiopulmonary bypass [28]. Therefore, the model is not completely representative, and it could be argued that it both underestimates and overestimates the response that could be anticipated in patients.

Determinations of dead space revealed a transient increase in the ventilatory deadspace directly after the administration of the shed blood surrogate. However, values returned to normal after 45 minutes. Other models of lipid macro-emboli have shown similar results [24]. However, in those studies the increase in deadspace lasted throughout the entire experiments. Our findings suggest that there is an acute phase in lipid micro-embolization combining highly elevated PVR and an increase in dead space, followed by a chronic phase with elevated PVR but normalized deadspace.

There was considerable inter-animal variation in response. All animals, with one exception, responded with an increase in pulmonary blood pressure and a decrease in arterial blood pressure. The animal that did not show a decrease in systemic pressure still had an increase in PVR. On the other hand, one animal went into circulatory shock and could not be resuscitated with high doses of epinephrine. This animal succumbed after 30 minutes, and was excluded from the analysis. Between the extremes, the responses varied. There was a clear association between the hemodynamic response and the change in ventilatory deadspace. However, no association was found between the hemodynamic response and the radioactivity in arterial blood. The pathophysiology leading to these different hemodynamic changes must surely be multifactorial. Intrapulmonary shunting, an open foramen ovale or, an individual susceptibility to lipid particles are some potential mechanisms.

In this study, we addressed issues of the potential danger of lipid micro-emboli in the pulmonary circulation, that have not been studied before. Our findings suggest partial lipid entrapment and occlusion in the capillaries of the lungs. The hemodynamic effects of this entrapment are transient, but strong, and are probably not directly transferable to the clinical setting, in which the infusion is slower. However, since we found effects of moderate duration on PVR, it appears that the acute occlusion is only one part of the pathophysiology, and that the delayed increase in PVR is also of clinical relevance. In addition, the constant passage of lipid particles through the lungs, which could be augmented by an increase in pulmonary artery pressure, subsequently led to arterial embolization of lipid particles. The significance of lipid emboli in organs such as the brain and the kidneys has been discussed previously, and the risk of serious organ dysfunction has been suggested [6,12-14]. The findings of this study further lends support to the importance of using strategies to eliminate lipid embolic material in shed mediastinal blood, by using cell saver techniques and/or filtration.

## Conclusion

The findings of this study bring into question the appropriateness of transfusing autologous blood containing lipid micro-emboli on the venous side. The suitability of this procedure should be especially questioned when the content of lipid micro-particles is high, when the volume of blood is large, or when there already is a compromised right ventricle or lung function.

## Competing interests

HB is a co inventor of technology which can be utilized for blood salvage and refining, and has a vested interest in that technology. PJ has received grants from Medtronics for studying mini extra corporeal perfusion systems.

## Authors' contributions

AE: key role in planning and performing the study, participated in the sample and data analysis. Co writer. IP: helped with the sample preparation and analysis. BB: key role in the execution of the animal experiments. PJ: planning and execution of the animal experiments. MD: Help with executing the animal experiments and performed the radioactivity analysis. HB: Planned the experiment and analysed data. Co writer. All authors read and approved the final manuscript.
